# Quality-Oriented Perceptual HEVC Based on the Spatiotemporal Saliency Detection Model

**DOI:** 10.3390/e21020165

**Published:** 2019-02-11

**Authors:** Xiantao Jiang, Tian Song, Daqi Zhu, Takafumi Katayama, Lu Wang

**Affiliations:** 1 Department of Information Engineering, Shanghai Maritime University, NO.1550, Haigang Ave. Shanghai 201306, China; 2 Department of Electrical and Electronics Engineering, Tokushima University, 2-24, Shinkura-cho, Tokushima 770-8501, Japan

**Keywords:** H.265/HEVC, perceptual video coding, video saliency model, bitrate reduction

## Abstract

Perceptual video coding (PVC) can provide a lower bitrate with the same visual quality compared with traditional H.265/high efficiency video coding (HEVC). In this work, a novel H.265/HEVC-compliant PVC framework is proposed based on the video saliency model. Firstly, both an effective and efficient spatiotemporal saliency model is used to generate a video saliency map. Secondly, a perceptual coding scheme is developed based on the saliency map. A saliency-based quantization control algorithm is proposed to reduce the bitrate. Finally, the simulation results demonstrate that the proposed perceptual coding scheme shows its superiority in objective and subjective tests, achieving up to a 9.46% bitrate reduction with negligible subjective and objective quality loss. The advantage of the proposed method is the high quality adapted for a high-definition video application.

## 1. Introduction

High efficiency video coding (HEVC), also known as H.265, is the latest video coding standard. It was released in 2013 and achieves an average bitrate reduction of 50% for fixed video quality compared with the H.264/AVC which is a block-oriented motion-compensation-based video compression standard. As regards the limited channel bandwidth and storage capacity, its coding efficiency needs further improvement. A perceptual model based on the human visual system (HVS) can be integrated into the video coding framework to achieve a low bitrate with a high perceptual quality.

Saliency refers to the indication of the probability of human attention according to the visual stimulus, and the term saliency often appears indistinguishably in two types of studies with different approaches; the “different outputs” approach and the “different evaluations” approach [1]. The first is a scientific study that aims to implement the psychophysical findings of human visual attention systems. This approach is referred to as a saliency map, and computes how much attention a pixel in a given image or video attracts, and then compares the output of the method with actual human gaze data. These outputs are often in the form of a two-dimensional map. The second type is a form of engineering studies, simply designed to extract meaningful objects. This approach is referred to as salient object detection, which aims to estimate regions in a given image that contain meaningful objects, and utilizes ground truth region labels for evaluation. Salient object detection is commonly interpreted in computer vision as a process that includes two stages: (a) detecting the most salient object and (b) segmenting the accurate regions of that object. These outputs are often in the form of a binary map.

Recently, perceptual video coding (PVC) has attracted lots of attention [2,3,4]. The perceptual models in previous works have been divided into four categories: the visual attention (VA) model, the region-of-interest (ROI) model, the visual sensitivity (VS) model, and the cross-modal attention (CMA) model. A detailed description of these methods is as follows.

The main idea, according to the visual attention model, is to encode the importance of different regions with bottom-up or top-down attention. Itti presents a biologically-motivated algorithm to select visually-salient regions for video compression [5]. Gou et al. propose a novel multiresolution spatiotemporal saliency detection model to calculate the spatiotemporal saliency map [6]. This method can improve the coding efficiency significantly and works in real time. Wei et al. present an H.265/HEVC-compliant perceptual video coding scheme based on visual saliency [7]. This method can reduce the bitrate significantly with negligible perceptual quality loss. However, in previous work, the video-based visual saliency model (VSM) has proved to be unsophisticated for use in video coding. The main idea of the ROI model is to encode an area around the predicted attention-grabbing regions with higher quality compared to other less visually important regions. Xu et al. present a region-of-interest based H.265/HEVC coding approach to improve the perceived visual quality [8]. Grois et al. define a dynamic transition region between the region-of-interest and backgrounds [9], then a complexity-aware adaptive spatial preprocessing scheme is presented for the efficient scalable video coding. However, the above methods do not make a distinction between the different parts of the non-moving region. The main idea, as regards the visual sensitivity model, is to improve the coding performance with kinds of visual signal distortion sensitivity differences in HVS [10]. However, the computation complexity is higher when using these models. The main idea of the cross-modal attention model is to encode using multimodal interaction mechanisms [11]. However, it is not applicable for no voice mixing of the video sequence. Recently, some innovative ideas have been developed to improve the performance of video coding. Ferroukhi et al. propose a method combining bandelets and the set partitioning in hierarchical trees (SPIHT) algorithm for medical video coding [12], and this method has shown good high performances in terms of visual quality and peak signal-to-noise rate (PSNR) at low bitrates. Rouis et al. present a novel approach for perceptually guiding the rate-distortion optimization (RDO) process in H.265/HEVC [13], and this proposed method demonstrates a superior rate-distortion (R-D) performance over a compared approach adopting a similar scheme. Moreover, the learning-based perceptual video coding methods are proposed to improve video quality [14,15]. However, the computation complexity of these methods is high.

In summary, the research related to PVC has made great progress in recent years, but there are deficiencies. For example, there is lack of effective saliency detection computing models for video coding applications. Image-based saliency detection has been extensively studied over the past decades. However, the research related to video-based saliency detection is much less explored. The existing saliency detection models only consider some of the characteristics of the image, and some of the temporal and context information is ignored. Moreover, most of the computation models are based on the pixel level. The computation complexity is too high, making it is unfavorable for real-time video applications.

In this paper, we proposed a new saliency detection model for video compression. Firstly, a novel saliency object detection model is proposed to generate a spatiotemporal saliency map. Secondly, the H.265/HEVC-compliant perceptual coding framework is developed based on the spatiotemporal saliency map. This paper has the following contributions: (1) a new superpixel-based salient object model is proposed which incorporates spatial and temporal features. (2) the proposed perceptual coding method achieves higher compression rates with a good balance between subjective and objective quality compared to the H.265/HEVC reference model.

## 2. Proposed Perceptual Video Coding Algorithm

### 2.1. System Overview

**Figure 1:**
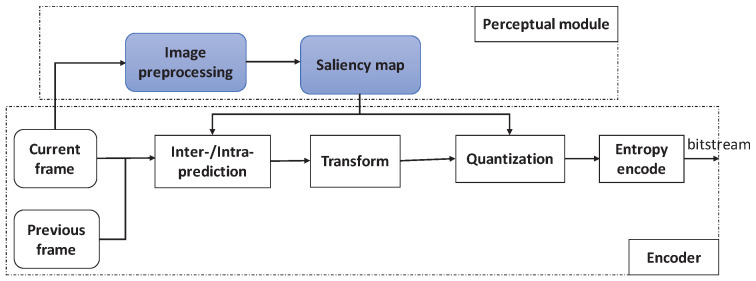
Proposed system framework of the perceptual video coding.

Figure 1 shows the framework of the saliency-based H.265/HEVC. This perceptual video coding framework includes two parts: the perceptual module and the encoder. The perceptual module is used to generate the saliency map using the spatiotemporal information. The saliency map is inputted so the encoder can determine the quantization parameters.

### 2.2. Video-Based Spatiotemporal Saliency Detection Model

For monitoring video, the moving object is the most attractive point of attention for a human. Meanwhile, there are the static features in the video. Thus, the spatiotemporal saliency map generated by the visual attention model and moving object detection model can be explored to guide the bit allocation for video coding.

For the input video signal, the spatial saliency and temporal saliency are computed using the spatiotemporal information, respectively. Therefore, according to a certain weight coefficient, the spatial and temporal saliency are merged into the final saliency map.

A good deal of research effort has already been devoted to saliency models for images. The image saliency models can be used for spatial saliency detection in each video frame. In this paper, the spatial saliency computation is based on the Markov chain (MC) saliency detection model developed by Lu et al. [16], which is both effective and efficient. The main details of the MC saliency algorithm are as follows:

- A single layer graph G(V,E) with superpixels is constructed, where *V* and *E* represent the nodes and edges of *G*, respectively. On the *G*, each node, including transient nodes and absorbing nodes, is connected to the transient nodes which neighbor it or share common boundaries with its neighboring nodes. Thus, the weight $w_{ij}$ of the edge $e_{ij}$ between adjacent nodes *i* and *j* is defined as
  (1) $$w_{ij}=e^{-\frac{\left|\right|x_{i}-x_{j}\left|\right|}{\sigma^{2}}}$$
  where $x_{i}$ and $x_{j}$ represent the mean of the two nodes in the CIELAB color space, and σ is a constant.
- The nodes are renumbered so that the first *t* nodes are transient nodes and the last *r* nodes are absorbing nodes, and the affinity matrix *A* and degree matrix *D* can be expressed with $w_{ij}$. Then, the transition matrix *P* on the sparsely connected graph can be calculated from matrix *A* and *D*. After that, using the Markov chain theory, the absorbed time for each transient state can be calculated based on the transition matrix *P*. Finally, the saliency map *S* can be generated by normalizing the absorbed time.
  The basic idea of the MC method is to detect the saliency using the time property in an absorbing Markov chain. The virtual boundary nodes are identified as absorbing nodes based on the prior boundary. The saliency is computed as the absorbed time to the absorbing nodes. On the basis of the MC saliency model, the spatial saliency value is represented as $S_{S}\left(i\right)$ for each pixel *i*.

Particularly for videos, the temporal information is a significant hint, and more context exists in the field of video saliency detection. In video saliency detection, the optical flow technique can detect motion accurately. $MV_{x}\left(x,y\right)$ and $MV_{y}\left(x,y\right)$ are represented as the horizontal and vertical motion vector by using the Lucas–Kanade (LK) algorithm [17]. Thus, the motion vector amplitude (MVA) MV(x,y) is calculated as

(2) $$MV\left(x,y\right)=\sqrt{MV_{x}{\left(x,y\right)}^{2}+MV_{y}{\left(x,y\right)}^{2}}.$$

Furthermore, the MVA is enhanced as

(3) $$S_{mv}\left(x,y\right)=\left\{\begin{array}{ll} \alpha \times MV(x,y)-\alpha \times \beta & \mathrm{if}\;MV(x,y)>\beta \\ 0 & \mathrm{otherwise} \end{array}\right.$$

where α and β are parameters, in this work, α=10 and β=2. Finally, the $S_{mv}\left(x,y\right)$ value is clipped to the [0, 255] range:

(4) $${\hat{S}}_{mv}\left(x,y\right)=\left\{\begin{array}{ll} 255 & \mathrm{if}\;S_{mv}\left(x,y\right)>255\\ S_{mv}\left(x,y\right) & \mathrm{others} \end{array}\right.$$

Then, the ${\hat{S}}_{mv}\left(x,y\right)$ can be assigned the temporal saliency value $S_{T}\left(i\right)$ for pixel i(x,y):

(5) $$S_{T}\left(i\right)={\hat{S}}_{mv}\left(x,y\right).$$

**Figure 2:**
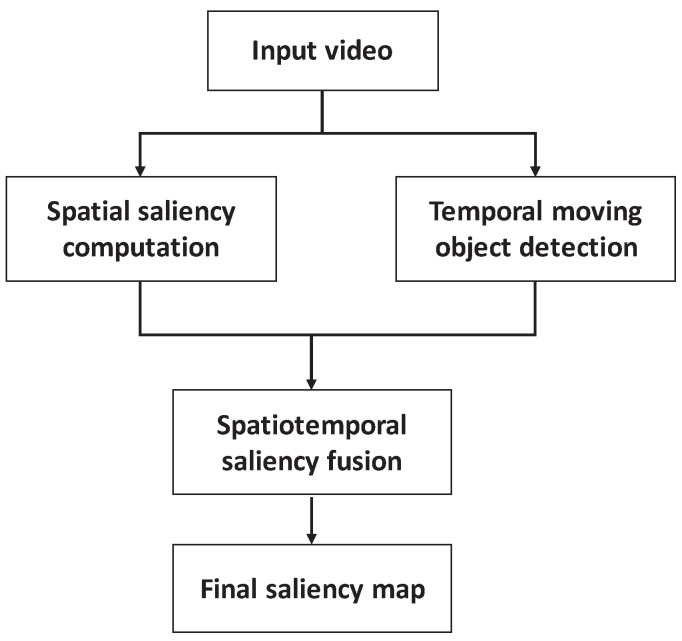
Spatiotemporal saliency fusion framework.

The temporal saliency reflects the dynamic characteristics of the video, while the spatial saliency reflects the static characteristics of the video. When the spatial and temporal saliency maps are constructed, we use them to get the final video saliency map. Figure 2 shows the block diagram of the spatiotemporal saliency fusion framework. For the input video signal, the spatial saliency and temporal saliency are computed by using the spatiotemporal information, respectively. Thus, according to a certain weight coefficient, the spatial and temporal saliency are merged into the final saliency map.

Different fusion methods can be utilized, and the linear fusion is used in this paper. The final video saliency map $S_{ST}\left(i\right)$ is generated by

(6) $$S_{ST}\left(i\right)=\left(1-\omega_{t}\right)\times S_{S}\left(i\right)+\omega_{t}\times S_{T}\left(i\right)$$

where $\omega_{t}$ is the weight of the temporal saliency, and $\left(1-\omega_{t}\right)$ is the weight of the spatial saliency. The weight $\omega_{t}$ is determined by the contribution of temporal characteristics in the spatiotemporal saliency. The best experimental combination that we obtained for Equation (6) was achieved for $\omega_{t}$ = 3/7 in this paper [18].

**Figure 3:**
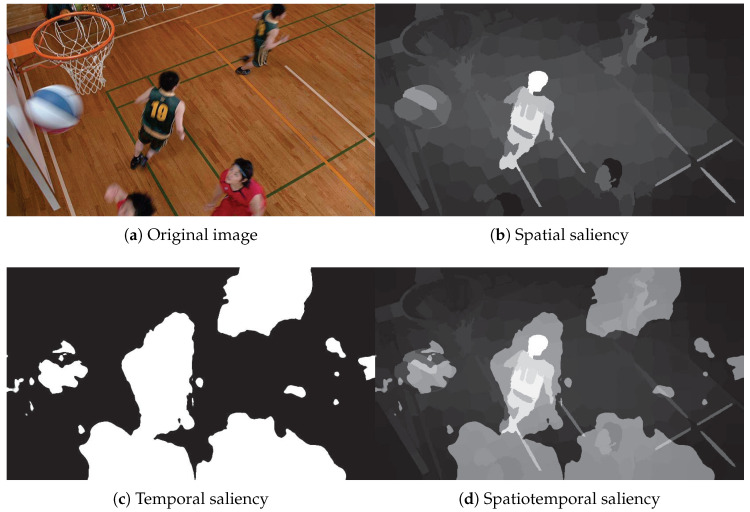
The saliency fusion of BasketballDrill sequence.

In the video, the moving objects are regarded as the salient region. Finally, the superposition of spatiotemporal saliency is taken as the final saliency with linear fusion. Figure 3d shows the spatiotemporal saliency for the third frame of the BasketballDrill sequence.

**Figure 4:**
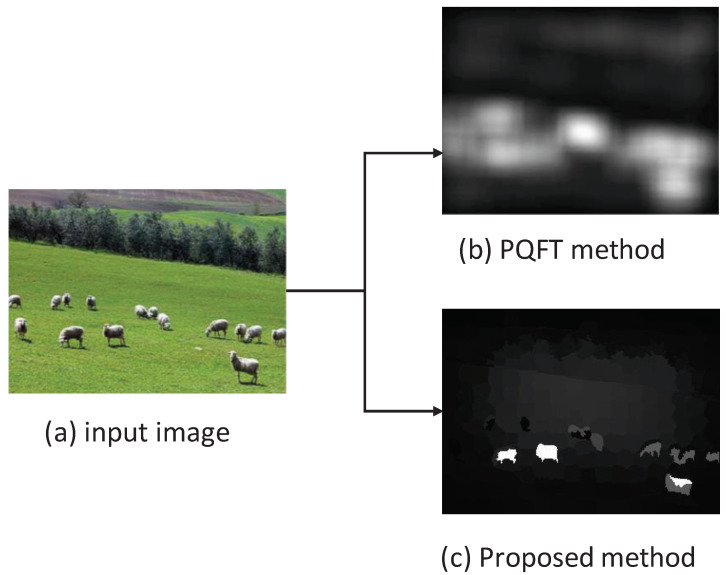
Saliency maps calculated by the phase spectrum of quaternion Fourier transform (PQFT) and proposed methods.

It is noted that this work is different from the existing method [6]. Firstly, the Markov chain-based saliency detection model in this work is a spatial domain approach, while the phase spectrum of quaternion Fourier transform (PQFT) model in the existing method [6] is a frequency domain one. However, the PQFT-based saliency model only highlights the boundaries of the salient object while the object interior still has a low saliency value. Secondly, the Markov chain-based saliency detection method in the proposed work can enhance the object interior significantly. Moreover, the proposed method is more effective in saliency detection. Figure 4 shows an example of the saliency maps calculated by PQFT and the proposed methods, and it was discovered that the proposed method can predict eye fixations better than the PQFT method.

Moreover, for good saliency detection, a model should meet at least the following two criteria: (a) Good detection: the probability of missing real salient regions and falsely marking the background as a salient region should be low. (b) High saliency value: the boundaries of salient regions and region interiors have a high saliency value. The advantages of the proposed work are as follows: (1) In this work, the optical flow technique is used to detect temporal saliency, which can detect moving objects precisely. (2) The Markov chain-based saliency detection method in the proposed work can enhance the region’s interior significantly. Thus, the generated saliency map with the proposed method can increase the coding performance.

### 2.3. Saliency-Based Quantization Control Algorithm

In the video, the moving objects are regarded as the salient region. Finally, the superposition of spatiotemporal saliency is taken as the final saliency with linear fusion.

In H.265/HEVC standards, the coding unit (CU) is a square region and is a node of the quad-tree partitioning of the coding tree units (CTU) [19]. The quad-tree partitioning structure allows recursive splitting into four equally sized nodes, and the minimum CU size is configured to be 32×32, 16×16, or 8×8 that of the luma samples. Coding unit tree is an algorithm for adaptive quantization parameters (QP). In order to control QP in the encoder, the saliency map can be partitioned into the block with a size of 64×64. Then, the block-based saliency average value ${\bar{S}}_{ST}\left(x,y\right)$ is calculated as

(7) $${\bar{S}}_{ST}\left(x,y\right)=\frac{\sum_{x=0}^{N}\sum_{y=0}^{N}S_{ST}\left(x,y\right)}{N^{2}}$$

where *N* is CU size, and $S_{ST}\left(x,y\right)$ is the saliency value of the pixel i(x,y) within a CU.

In H.265/HEVC standards, a QP is used to determine the quantization step, the setting range of QP is from 0 to 51. In order to associate the block-based saliency value with QP, the saliency value can be normalized to the range [0, 3]. In this paper, the min–max normalization method is used to generate the saliency level for the block-based saliency average value ${\bar{S}}_{ST}\left(x,y\right)$:

(8) $$\Delta S=3\times \frac{{\bar{S}}_{ST}\left(x,y\right)-S_{min}}{S_{max}-S_{min}}$$

where ΔS is the calculated saliency level, and $S_{max}$ and $S_{min}$ are the maximums and minimums of ${\bar{S}}_{ST}\left(x,y\right)$, respectively.

When the ΔS value is high, the corresponding image can be encoded as a foreground. In contrast, when the ΔS value is low, the corresponding image can be encoded as a background. In the perceptual video coding process, the foreground needs to increase the allocated bitrate resources, while the background needs to reduce the allocated bitrate resources. In video coding, QP is used to adjust distortion. When the QP is larger, the image distortion is higher. Therefore, a high QP is used for the foreground coding, and a low QP is used for the background coding. On the basis of the saliency level ΔS, the setting quantized parameters ($QP_{set}$) can be dynamically adjusted in the encoding process. When $QP_{def}$ is the default quantized parameter, and the $QP_{def}$ has four values (22, 27, 32, and 37) in H.265/HEVC reference software. Thus, the saliency-based quantization control algorithm is shown in Algorithm 1. By using the proposed method, more image distortion is introduced to CUs with a low saliency while less to CUs with a high saliency.

**Algorithm 1:** Saliency-based quantization control algorithm

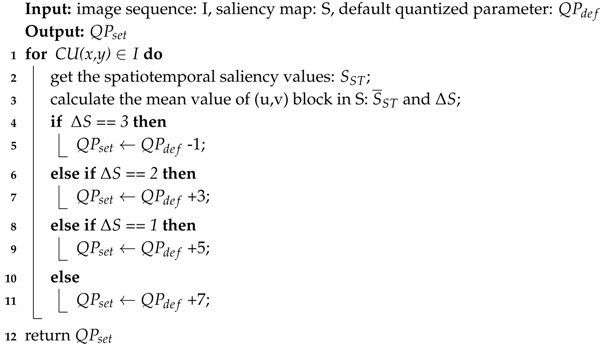



## 3. Experiment Results

The proposed algorithm is implemented and verified based on the H.265/HEVC reference model HM 16.0. The quantization parameters are set to 22, 27, 32, and 37, respectively. The configuration profile is low-delay (LD).

The performance of the proposed algorithm is evaluated by the average bitrate saving (BS) and Bjontegarrd Delta PSNR (BD-PSNR) according to Reference [20]. The BS is calculated as

(9) $$BS\left(\%\right)=\frac{1}{4}\sum_{i=1}^{4}\frac{BR_{HM}\left(QP_{i}\right)-BR_{pro}\left(QP_{i}\right)}{BR_{HM}\left(QP_{i}\right)}\times 100\%$$

where $BR_{HM}\left(QP_{i}\right)$ and $BR_{pro}\left(QP_{i}\right)$ are the bitrates using the H.265/HEVC reference software and the proposed method with different $QP_{i}$.

The double stimulus continuous quality scale (DSCQS) method is used for subjective evaluation [21]. The subjects are presented with pairs of video sequences, where the first sequence is the H.265/HEVC reference video and the second sequence is the video with the proposed method. Secondly, a total of 24 naive viewers take part in the test campaign, and all viewers are screened for the correct level of visual acuity and color vision. Thirdly, viewers are expected to mark their visual quality score on an answer sheet with a quality rating scale over a defined scale that is defined in Table 1.

The subjective evaluation of video quality is evaluated by the difference mean opinion score (MOS) average values Delta MOS (D-MOS), which is calculated as

(10) $$\mathrm{D-MOS}=\frac{1}{4}\sum_{i=1}^{4}\left(MOS_{pro}\left(QP_{i}\right)-MOS_{HM}\left(QP_{i}\right)\right)$$

where $MOS_{pro}\left(QP_{i}\right)$ and $MOS_{HM}\left(QP_{i}\right)$ are the measured MOS values from the sequence encoded by the proposed method and the H.265/HEVC reference software HM 16.0 with different $QP_{i}$, respectively.

In the ablation study, the results of spatial-only, temporal-only, and the proposed spatiotemporal saliency-based quantization control scenarios are shown in Table 2. From the experimental results, it can be seen that the average bitrate saving and Bjontegarrd Delta PSNR loss are 6.51%, 0.059 dB, and 6.09%, 0.053 dB for the spatial-only, temporal-only scenarios, respectively. For the proposed spatiotemporal method, the bitrate can be reduced by 5.22% while the Bjontegarrd Delta PSNR loss is only 0.046 dB on average. Thus, the objective distortion of the proposed method is smaller compared with the spatial-only and temporal-only scenarios.

For our experiments, we selected four test sequences of different resolutions. The four test sequences were also selected by considering the diversity of spatial characteristics and temporal characteristics. The four test sequences were the ‘ParkScene’ sequence with a resolution of 1920×1080, the ‘Vidyo1’ sequence with a resolution of 1280×720, the ‘BasketballDrill’ sequence with a resolution of 832×480, and the ‘BasketballPass’ sequence with a resolution of 416×240. The test results of the proposed method are shown in Table 3, compared to H.265/HEVC reference software with four QP values of 22, 27, 32, and 37, It shows that the proposed PVC method can achieve 1.22%–8.84% bitrate reduction for the test sequences, where $Bitrate-Reduction\left(\%\right)=\frac{HM16.0\;Bitrates-Proposed\;Bitrates}{HM16.0\;Bitrates}\times 100\%$. It is also observed in Table 3 that the bitrate reduction values are usually decreased as the QP value increases for the proposed method. This is because the distortions introduced by quantization errors are high enough at high QP values. It is noted in Table 3 that the average time saving is 0.16% for the test sequences ($Time-Saving\left(\%\right)=\frac{HM16.0\;Encoding\;Time-Proposed\;Encoding\;Time}{HM16.0\;Encoding\;Time}\times 100\%$), compared to the H.265/HEVC reference software. Thus, the proposed method is effective, and the computational cost of the proposed method is almost the same as the H.265/HEVC reference software.

**Figure 5:**
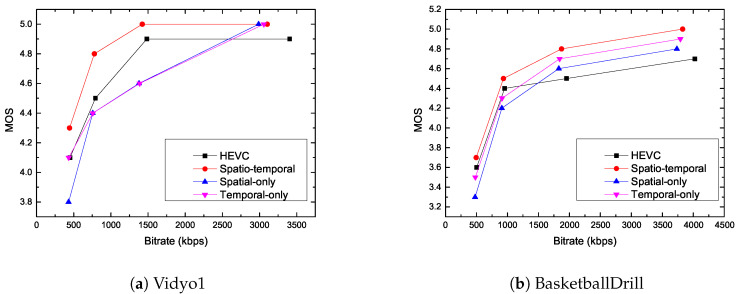
Rate mean opinion score (MOS) curves for quantization control algorithm.

**Figure 6:**
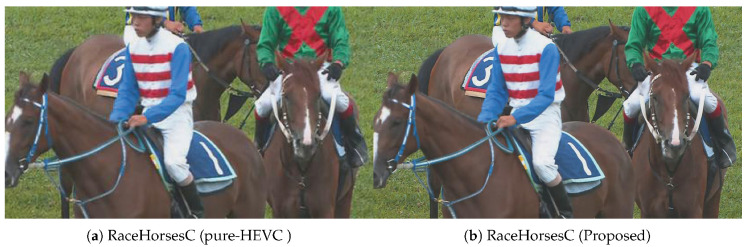
Visual quality comparison between the pure high efficiency video coding (HEVC) and the proposed method.

When the Delta MOS values are smaller, the subjective qualities are closer to the original H.265/HEVC reference software. The fifth, eighth, and eleventh columns in Table 2 show the visual quality for the spatial-only, temporal-only, and spatiotemporal scenarios. It can be seen from the table that the average absolute Delta MOS value of the proposed method is even smaller than 0.1. Figure 5 shows the typical example of MOS curve for Vidyo1 and BasketballDrill sequences among the spatial-only, temporal-only, spatio-temporal methods, and the H.265/HEVC reference model. From these figures, it can be seen that the proposed method shows visual quality improvements over the H.265/HEVC reference model, spatiotemporal methods, and temporal-only scenarios. Furthermore, when QP is set to 37, Figure 6 shows the difference between the image of pure-HEVC and the image of the proposed reconstructed video for RaceHorsesC sequence. As can be seen from the graphic, at the low bit-rate, the visual quality of the proposed method is better than the visual quality of pure-HEVC.

Furthermore, the comparative results with previous work are shown in Table 4, with QP set to 32. For Bae’s work [10], the average bitrate saving and Delta MOS are 1.4%, 0.3, respectively. In contrast, the average bitrate can be reduced by 3.9% and the Delta MOS is 0.2 for the proposed scheme. Thus, the performance of the proposed method is superior to Bae’s work.

In summary, from the objective and subjective test results, the proposed perceptual video coding scheme can achieve higher compression rates with the balance between subjective and objective quality compared to the H.265/HEVC reference model.

## 4. Conclusions

In this paper, we proposed a novel perceptual video coding scheme based on the spatiotemporal saliency model. Simulation results demonstrate that the proposed perceptual coding scheme can save of 5.22% bitrate, while the Bjontegarrd Delta PSNR is only 0.046 dB on average. Moreover, the average absolute Delta MOS value of the proposed method is even smaller than 0.1.

## Figures and Tables

**Table 1:** Scale.

Scale	MOS
Very annoying	1
Annoying	2
Slightly annoying	3
Perception but not annoying	4
Imperception	5

**Table 2:** The results of quantization control algorithm.

		Spatial-Only			Temporal-Only			Spatio-Temporal (Proposed)		
Class	Sequence	BS(%)	BD-PSNR(dB)	D-MOS	BS(%)	BD-PSNR(dB)	D-MOS	BS(%)	BD-PSNR(dB)	D-MOS
1920×1080	**ParkScene**	4.66	−0.146	−0.059	4.13	−0.173	0.029	4.75	−0.139	0.206
	**Cactus**	6.35	−0.142	−0.323	5.80	−0.129	−0.235	5.45	−0.140	0.030
	**BQTerrace**	11.01	−0.155	−0.294	9.56	−0.133	−0.206	9.46	−0.148	0.073
1280×720	**Vidyo1**	7.01	−0.060	−0.147	6.20	−0.010	−0.059	4.06	−0.048	0.162
	**Vidyo3**	9.64	−0.049	−0.162	7.59	−0.001	−0.073	6.21	−0.057	0.118
	**Vidyo4**	7.66	−0.031	−0.088	6.47	−0.015	−0.015	5.14	−0.025	0.162
**High Res.**	**Average**	7.72	−0.097	−0.179	6.63	−0.077	−0.093	5.85	−0.093	0.125
832×480	**BasketballDrill**	5.78	−0.017	0.029	4.94	−0.047	0.117	3.06	−0.003	0.206
	**BQMall**	7.39	−0.005	−0.177	6.59	0.012	−0.177	5.81	0.016	0.132
	**PartyScene**	6.13	−0.052	−0.236	6.08	−0.068	−0.147	4.80	−0.044	0.103
	**RaceHorsesC**	0.99	−0.115	−0.192	2.05	−0.152	−0.103	3.31	−0.094	0.044
416×240	**BasketballPass**	6.27	0.027	−0.133	5.98	0.020	−0.045	5.17	0.058	0.014
	**BQSquare**	7.08	0.042	−0.238	8.34	0.043	−0.177	5.75	0.060	−0.059
	**BlowingBubbles**	7.40	−0.057	−0.323	7.49	−0.033	−0.235	6.08	−0.031	−0.191
	**RaceHorses**	3.79	−0.070	−0.176	4.03	−0.059	−0.088	3.98	−0.044	−0.015
**Low Res.**	**Average**	5.60	−0.031	−0.181	5.69	−0.036	−0.107	4.74	−0.010	0.029
**Average**		6.51	−0.059	−0.180	6.09	−0.053	−0.101	5.22	−0.046	0.070

**Table 3:** The performance comparison with the H.265/HEVC reference software.

		PSNR (dB)		Bitrates (kbps)			
Sequence (Size)	QP	HM16.0	Proposed	HM16.0	Proposed	Bitrate−Reduction (%)	Time−Saving (%)
ParkScene (1920×1080)	22	40.02	39.58	10,462.13	9805.68	6.27	2.73
	27	37.33	37.00	4492.62	4270.77	4.94	1.04
	32	34.65	34.41	2034.98	1955.44	3.91	−1.37
	37	32.16	31.98	924.67	888.67	3.89	−1.50
Vidyo1 (1280×720)	22	43.34	43.05	3405.15	3104.26	8.84	0.18
	27	41.25	41.07	1483.55	1422.43	4.12	−1.71
	32	38.92	38.82	791.84	778.56	1.68	−2.81
	37	36.21	36.12	450.40	443.17	1.61	−3.96
BasketballDrill (832×480)	22	40.80	40.56	4025.28	3828.77	4.88	4.67
	27	37.66	37.49	1953.63	1871.79	4.19	0.80
	32	34.72	34.63	954.91	936.29	1.95	−1.15
	37	32.23	32.17	501.79	495.65	1.22	−1.30
BasketballPass (416×240)	22	41.44	41.09	1086.85	1003.60	7.66	2.02
	27	37.82	37.58	560.75	527.07	6.01	2.44
	32	34.47	34.33	282.05	272.08	3.54	1.23
	37	31.48	31.36	145.73	140.69	3.46	1.21
**Average**						4.26	0.16

**Table 4:** Comparisons of rate and Delta MOS values (QP = 32).

	Bae [10]		Proposed	
Sequence	BS(%)	D-MOS	BS(%)	D-MOS
BQTerrace	3.6	0.9	5.9	0.1
Catus	0.8	0.0	2.2	0.0
ParkScene	0.8	−0.3	3.3	0.1
BQMall	0.9	0.2	4.2	0.4
Average	1.4	0.3	3.9	0.2
